# Transcriptome analysis reveals overlap in fusion genes in a phase I clinical cohort of TNBC and HGSOC patients treated with buparlisib and olaparib

**DOI:** 10.1007/s00432-019-03078-9

**Published:** 2019-11-19

**Authors:** Julia Eismann, Yujing J. Heng, Johannes M. Waldschmidt, Ioannis S. Vlachos, Kathryn P. Gray, Ursula A. Matulonis, Panagiotis A. Konstantinopoulos, Charles J. Murphy, Sheida Nabavi, Gerburg M. Wulf

**Affiliations:** 1 Department of Hematology/Oncology, Beth Israel Deaconess Medical Center, Boston, MA USA; 2 Harvard Medical School, Boston, MA USA; 3 Department of Obstetrics and Gynecology, University Medical Center Freiburg, Freiburg, Germany; 4 Department of Pathology, Beth Israel Deaconess Medical Center, Boston, MA USA; 5 Department of Medical Oncology, Dana-Farber Cancer Institute, Boston, MA USA; 6 Biostatic Core, Dana-Farber Cancer Institute, Boston, MA USA; 7 Institute for Computational Biomedicine, Weill Cornell Medical College, New York, NY USA; 8 Department of Computer Science and Engineering, Institute of System Genomics, University of Connecticut, Storrs, USA; 9 Broad Institute of MIT and Harvard, Cambridge, MA USA

**Keywords:** Fusion gene, Breast cancer, Ovarian cancer, Genomic profiling, RNA-seq

## Abstract

**Purpose:**

Fusion genes can be therapeutically relevant if they result in constitutive activation of oncogenes or repression of tumor suppressors. However, the prevalence and role of fusion genes in female cancers remain largely unexplored. Here, we investigate the fusion gene landscape in triple-negative breast cancer (TNBC) and high-grade serous ovarian cancer (HGSOC), two subtypes of female cancers with high molecular similarity but limited treatment options at present.

**Methods:**

RNA-seq was utilized to identify fusion genes in a cohort of 18 TNBC and HGSOC patients treated with the PI3K inhibitor buparlisib and the PARP inhibitor olaparib in a phase I clinical trial (NCT01623349). Differential gene expression analysis was performed to assess the function of fusion genes in silico. Finally, these findings were correlated with the reported clinical outcomes.

**Results:**

A total of 156 fusion genes was detected, whereof 44/156 (28%) events occurred in more than one patient. Low recurrence across samples indicated that the majority of fusion genes were private passenger events. The long non-coding RNA *MALAT1* was involved in 97/156 (62%) fusion genes, followed in prevalence by *MUC16, FOXP1, WWOX* and *XIST.* Gene expression of *FOXP1* was significantly elevated in patients with vs. without *FOXP1* fusion (*P*= 0.02). From a clinical perspective, *FOXP1* fusions were associated with a favorable overall survival.

**Conclusions:**

In summary, this study provides the first characterization of fusion genes in a cohort of TNBC and HGSOC patients. An improved mechanistic understanding of fusion genes will support the future identification of innovative therapeutic approaches for these challenging diseases.

**Electronic supplementary material:**

The online version of this article (10.1007/s00432-019-03078-9) contains supplementary material, which is available to authorized users.

## Introduction

Breast cancer is the most frequently diagnosed female cancer in the United States with a lifetime risk of 12% and an expected number of 268,600 new cases and 41,760 deaths in 2019 (American Cancer Society 2019a). Triple-negative breast cancer (TNBC) occurs in 12–17% of breast cancer and is a subtype that does not express estrogen receptor (ER), progesterone receptor (PR) or human epidermal growth factor receptor 2 (HER2) (Foulkes et al. 2010). TNBC is an aggressive disease with high rates of metastasis and/or recurrence and has worse prognosis compared to HER2 + and ER + subtypes. Since TNBC patients do not respond to hormonal treatment or HER2-directed therapy, treatment options are restricted to chemotherapy such as platinum/taxane or alkylating agents.

Ovarian cancer represents the fifth most frequently diagnosed female cancer in the United States with a lifetime risk of 1.3% and an estimated number of 22,530 new cases and 13,980 deaths in 2019 (American Cancer Society 2019b). Advanced stage high-grade serous ovarian cancer (HGSOC) occurs in 70% of these patients (Koonings et al. 1989). Standard treatment for HGSOC consists of surgery followed by platinum/taxane chemotherapy. Despite these measures, the prognosis remains grim. 25% of HGSOC recur within the first 6 months after treatment and a 5-year overall survival (OS) of 31% has been reported (Jemal et al. 2009).

On the genomic level, TNBC and HGSOC share similar alterations, including widespread genomic instability, p53 mutations, deficiency in DNA damage repair and homologous recombination as well as PI3-kinase pathway activation (Cancer Genome Atlas Network 2012; Bell et al. 2011). Prior analyses indicate that PI3-kinase suppression can further inhibit homologous DNA double-strand break repair by impairing the non-oxidative pentose phosphate pathway (PPP) which physiologically provides ribose-5-phosphate for nucleoside synthesis (Hu et al. 2016). This, along with preclinical data derived from a PDX mouse model (Juvekar et al. 2012), provides the rationale to examine buparlisib and olaparib as a dual strategy to block PI3-kinase signaling and DNA repair in both diseases.

We recently assessed the safety and efficacy of this approach in the context of a multicenter phase I trial for patients with recurrent TNBC and HGSOC. Clinical responses in this study were observed in 28% of TNBC and in 29% of HGSOC patients (Matulonis et al. 2017).

Over the past years, the emergence of next-generation sequencing (NGS) technologies has dramatically transformed our ability to comprehensively assess genomic features and their impact on cancer pathogenesis and outcome. Amongst others, NGS allows for the study of fusion genes which result from translocations, interstitial deletions or chromosomal inversions of two separate genes (Mertens et al. 2015).

These genes can be therapeutically relevant if they result in constitutive activation of fused oncogenes or repression of fused tumor suppressor genes. This is exemplified by the oncogenic *BCR*–*ABL1* fusion gene, which was first detected in patients with chronic myelogenous leukemia (CML). The inhibition of BCR–ABL1 by the tyrosine kinase inhibitor imatinib led to dramatically improved molecular responses and survival of CML patients (Roy et al. 2006). Fusion genes have also been reported in solid tumors, e.g., *TMPRSS2*–*ERG* in prostate cancer, *EML4*–*ALK* in lung cancer and *EWS*–*FL1* in Ewing’s sarcoma (Tomlins et al. 2005; Soda et al. 2007; Owen et al. 2008). In female cancers, the *ETV6*–*NTRK3* fusion gene has previously been described, but its prevalence is exclusively limited to secretory breast cancer in which it may be detected in > 90% of all cases (Tognon et al. 2002). The prevalence and therapeutic potential of fusion genes in TNBC and HGSOC remain unexplored.

In the present study, we investigate the fusion gene landscape in the transcriptome of 18 TNBC and HGSOC patients who were treated with buparlisib and olaparib in the aforementioned phase I trial using RNA sequencing. We identify fused genes, assess in silico whether the resulting product is still functional, and investigate whether fusion genes result in differential expression of the respective genes involved. We correlate our findings with the reported clinical outcomes and evaluate if fusion genes are associated with clinical outcomes.

## Materials and methods

### Patient and tumor samples

The primary objective of this study was to investigate the fusion gene landscape and the effect of fusions on the function and expression of each partner gene. Samples were derived from a subset of patients from a phase I trial (NCT01623349) (Matulonis et al. 2017). The trial tested the safety and efficacy of the PI3K inhibitor buparlisib and the PARP inhibitor olaparib in a 3 + 3 dose-escalation design in patients with TNBC (*n *= 24) and HGSOC (*n *= 46). Out of 70 enrolled patients, formalin-fixed paraffin-embedded primary tumor biopsies were available for nine TNBC and nine HGSOC patients (Table 1).

**Table 1:** Patient characteristics (*n *= 18)

Variables	All (*n *= 18)	TNBC (*n *= 9)	HGSOC (*n *= 9)
Age at diagnosis			
Years [mean ± SEM (range)]	56 ± 2.5 (36–72)	55 ± 3.4 (36–70)	56 ± 3.7 (38–72)
Age at inclusion			
Years [mean ± SEM (range)]	60 ± 2.3 (38–78)	59 ± 3.2 (38–70)	60 ± 3.6 (43–78)
Race			
White	100%	100%	100%
Ethnicity			
Hispanic or Latino	2 (11.1%)	1 (11.1%)	1 (11.1%)
Non-Hispanic	15 (83.3%)	7 (77.8%)	8 (88.9%)
Unknown	1 (5.6%)	1 (11.1%)	
*BRCA* status			
*BRCA* wild type	5 (28%)	3 (33%)	2 (22%)
*BRCA 1* mutant	5 (28%)	1 (11%)	4 (44%)
*BRCA 2* mutant	5 (28%)	3 (33%)	2 (22%)
Unknown	3 (17%)	2 (22%)	1 (11%)
Platinum status			
Platinum resistant	7 (38.9%)	1 (11.1%)	6 (66.7%)
Platinum sensitive	5 (27.8%)	2 (22.2%)	3 (33.3%)
Unknown	6 (33.3%)	6 (66.7%)	
Stage			
I	2 (11.1%)	2 (22.2%)	
II	4 (22.2%)	3 (33.3%)	1 (11.1%)
III	9 (50.0%)	4 (44.4%)	5 (55.6%)
IV	3 (16.7%)		3 (33.3%)
Histology			
Adenocarcinoma	4 (22.2%)	4 (44.4%)	
Papillary serous	8 (44.4%)		8 (88.9%)
Transitional	1 (5.6%)		1 (11.1%)
Others	5 (27.8%)	5 (55.6%)	
Clinical grade			
Moderately differentiated	3 (16.7%)	3 (33.3%)	
Poorly differentiated	15 (83.3%)	6 (66.7%)	9 (100%)
Progression-free survival (PFS)			
Months [mean ± SEM (range)]	13.7 ± 3.2	10.2 ± 1.9	18.2 ± 6.9
	(1.9–55.7)	(2.8–19.2)	(1.9–55.7)
Reason for discontinuation			
Progression by RECIST 1.1	16 (88.9%)	9 (100%)	7 (77.8%)
Unacceptable toxicity	2 (11.1%)		2 (22.2%)
Overall survival			
Reached^a^	12 (67%)	6 (67%)	6 (67%)
Not reached	6 (33%)	3 (33%)	3 (33%)
Overall survival^a^			
Years (mean, range)	6.3 ± 1.2 (0.8–17.2)	5.8 ± 2.4 (0.8–17.2)	6.8 ± 0.6 (4.5–9.0)

^a^Median overall survival was only calculated for the 12 patients (6 TNBC, 6 HGSOC) that had reached EoT already, as indicated above

### RNA extraction and transcriptome sequencing

RNA was extracted using the Qiagen RNeasy FFPE kit (Germantown, MD) adhering to the manufacturer’s instructions. Library preparation was performed with the TruSeq RNA Access Library Prep Kit (Illumina, San Diego, CA). Paired-end, 75-bp reads were generated on a NextSeq 500 using a High Output, 150 cycle kit with v2 chemistry (Illumina).

### Quality control

Data output from sequencing was generated as raw test files in FASTQ format. All FASTQ files passed quality control using FASTQC v0.11.2 (http://www.bioinformatics.babraham.ac.uk/projects/fastqc). Splice-aware genome alignment was performed using the STAR aligner tool v020201 (Dobin et al. 2013). Reads were mapped to the human genome GRCh37 (hg19). Gene expression at transcript-level resolution was calculated using RSEM v.1.2.31 (Li and Dewey 2011). Gene annotations were derived from the Ensembl database (Ensembl Archive Release 94, October 2018).

### Detection of fusion transcripts

Fusion genes were detected using FusionCatcher v099.6a.b (Nicorici et al. 2014). Next, to reduce false positives, we applied a stepwise filtering process and excluded read-through fusions, fusions described as “non-tumor” or “non-cancer-tissue”, fusions that consisted of two adjacent fusion partners (annotated as “distance 1000 bp”, “distance 100 kbp”, “distance 10 kbp”), fusions with ribosomal or mitochondrial genes, fusions that involved immunoglobulin genes, fusions with identical breakpoints in more than three patients, fusions with < 2 spanning pairs and unique reads, pseudogenes and fusions without annotated genes (19). This resulted in the exclusion of 374 (47%) fusion genes with a high likelihood of being false positive (Supplementary Table 1). Another 262 fusions (33%) were removed as they occurred multiple times with the same breakpoints across patients. The remaining 156 events were evaluated in silico for the predicted effect of each fusion. Supplementary Table 2 provides more information about their expected function: 109 fusions (68%) had one gene partner with a non-coding DNA sequence (non-CDS). Nineteen fusions (12%) involved a partner gene with a truncated coding region. Ten fusions (6%) were predicted to be in-frame. Three fusions (2%) were out-of-frame and seven fusions (4%) were either intronic or CDS-complete. The function of eight fusion genes (5%) could not be predicted.

### Differential gene expression

Differential gene expression analyses between patient subgroups were performed using *DESeq2* (v1.22.2) (Love et al. 2014), while control of type I error in multiple hypothesis testing was done by calculating *q* value using the *q* value package (v2.14.1) from R. Gene fusion rearrangements were displayed by Circus plots generated from the copy number package v.1.22.0 as previously described (Krzywinski et al. 2009; Nilsen et al. 2012). Unless otherwise stated, all analyses were performed in R (version 3.5.1).

### Statistical analysis

Data are presented as of November 26, 2018. Continuous variables are presented as mean ± standard error of the mean (SEM). Overall survival (OS) was calculated as the time from first diagnosis until death. Progression-free survival (PFS) was calculated as the time from the beginning of study participation to the first observation of progressive disease. Two out of 18 patients could not be evaluated for their PFS as they were removed from the trial due to drug toxicity. Calculations of mean OS and PFS were performed using GraphPad Prism (Version 7.0e, La Jolla, CA, USA). For gene fusion correlation analyses to clinical endpoints, median OS ± 95% confidence interval [CI] was calculated by Kaplan–Meier estimators and tested with log-rank (Mantel–Cox) test using *ggplot2* (v.3.1.1) and *survival* (v. 2.44-1.1) from R. For this purpose, subjects who were still alive or lost to follow-up were included as censored observations. OS and PFS comparisons between groups were calculated by Kaplan–Meier analyses using *ggfortify* from R (v.0.4.6). For all other two-group comparisons, the Wilcoxon–Mann–Whitney test was used. The Kruskal–Wallis test was applied for the testing between three groups or more. A *P* value of *P *< 0.05 was considered to be statistically significant and levels of significance were marked as follows *P < 0.05, **P < 0.01 and ***P < 0.001. Graphical representations of the data are given as boxplots.

## Results

TNBC (*n *= 9) and HGSOC (*n *= 9) patients were of similar age at diagnosis, ethnicity and *BRCA* status (Table 1). In the TNBC cohort, four patients had adenocarcinoma and the histological subtypes of the other five TNBC patients could not be further specified. Eight of the HGSOC patients had papillary serous carcinoma and one had transitional carcinoma. A trend was noted toward to early UICC (Union of International Cancer Control) stage tumors in the TNBC subgroup whereas advanced disease stage tumors dominated in the HGSOC subcohort. PFS was measured at a mean of 10.2 ± 1.9 (± SEM) months for TNBC patients and at a mean of 18.2 ± 6.9 (± SEM) months for HGSOC patients. Mean OS was calculated for six TNBC and six HGSOC patients as available at the time of data collection. Mean OS in the TNBC cohort was 5.8 ± 2.4 (± SEM) years and 6.8 ± 0.6 (± SEM) years in the HGSOC subgroup. Intergroup comparison did not reveal a significant difference for PFS (*P *= 0.23) and OS (*P *= 0.71) between TNBC and HGSOC patients.

### Fusion landscape of TNBC and HGSOC patients

**Fig. 1:**
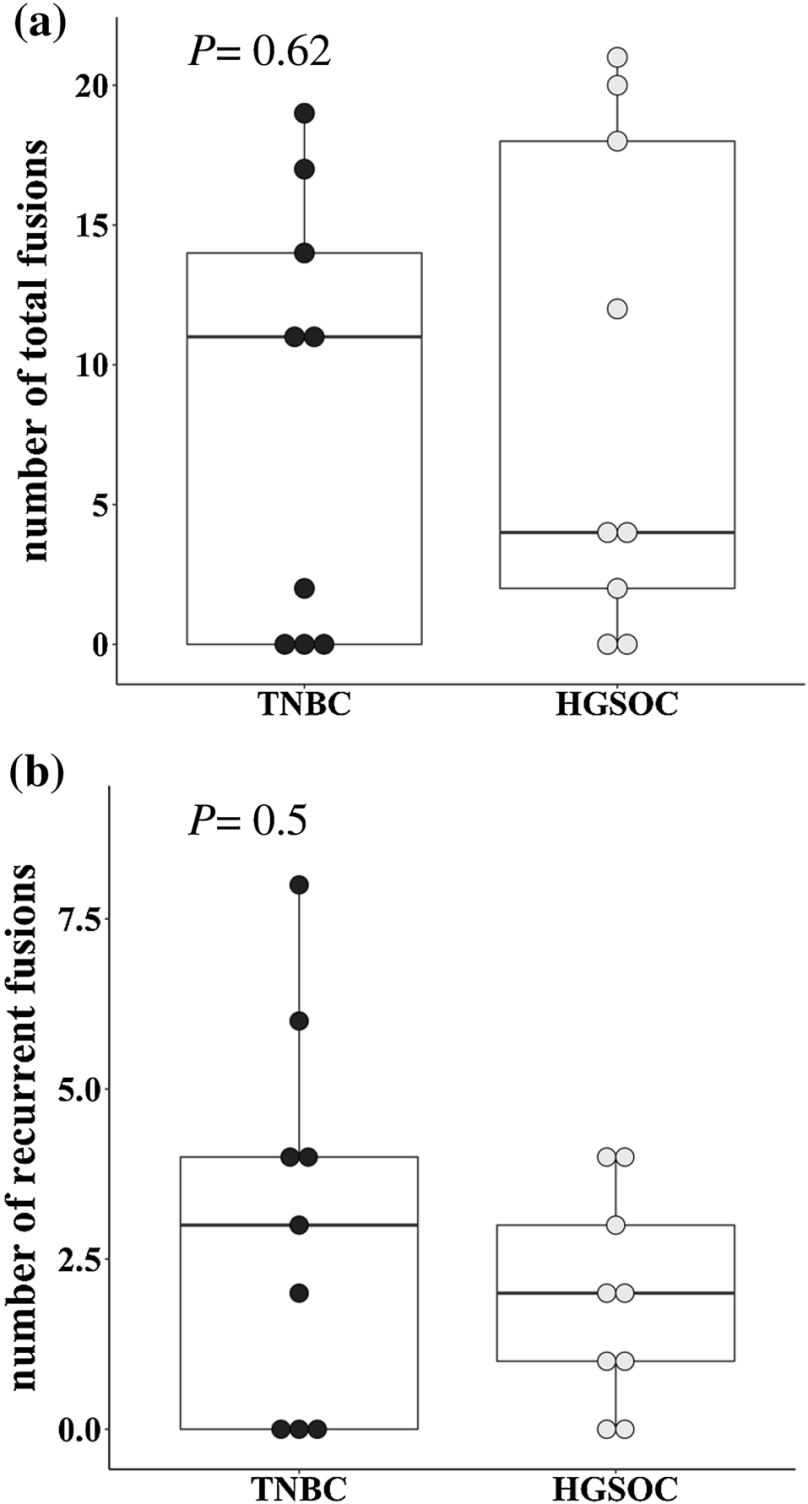
Fusion landscape across TNBC and HGSOC. Box plots were used to illustrate the number of fusions per patient. No significant difference between TNBC and HGSOC patients was noted for the number of total fusions (**a** Mann–Whitney–Wilcoxon *P *= 0.62) and the number of recurrent fusions (**b** Mann–Whitney–Wilcoxon *P *= 0.5)

The number of fusion genes in the entire cohort ranged from 0 and 21 per patient, with a mean of 8.7 ± 1.9 (± SEM) (Table 2). No fusion event was detected in three out of nine (33%) TNBC patients [mean 8.2 ± 2.6 (± SEM)] and two out of nine (22%) HGSOC patients [mean 9.1 ± 2.9 (± SEM)]. There was no difference in the number of fusion transcripts between TNBC and HGSOC patients (Fig. 1a, *P *= 0.62) and no association between the number of fusions and (1) age (*P *= 0.63), (2) UICC stage (*P *= 0.95), (3) clinical grade (*P *= 0.86) or (4) *BRCA* mutation status (*P *= 0.28, Supplementary Fig. 1).

**Table 2:** Number of patients with fusions across triple-negative breast cancer (TNBC, *n *= 9) and high-grade serous ovarian cancer (HGSOC, *n *= 9)

Fusions per entity	*n*	% of total	Mean ± SEM (range)
All patients	18	100	
No fusions detected	5	28	8.7 ± 1.9 (0–21)
Fusions detected	13	72	
TNBC	9	100	
No fusions detected	3	33	8.2 ± 2.6 (0–19)
Fusions detected	6	66	
HGSOC	9	100	
No fusions detected	2	22	9.1 ± 2.9 (0–21)
Fusions detected	7	78

### Only a fraction of fusion genes is recurrent

**Fig. 2:**
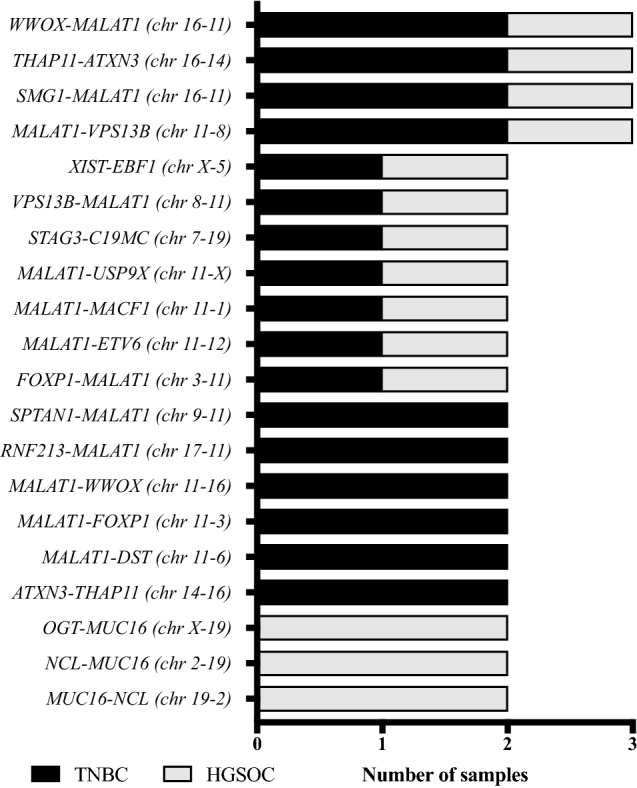
Frequency of the most prevalent recurrent fusion gene combinations across TNBC and HGSOC. Bar plots illustrating the most prevalent recurrent fusion gene combinations across both diseases. The gene location on its respective chromosome is given in brackets. *MALAT1* was involved in the majority of fusion transcripts. Three fusions genes involving *MUC16* were detected in more than one patient but were exclusively limited to HGSOC patients and were absent in TNBC patients. Eleven out of 20 (55%) of the most common fusion combinations could be observed in TNBC as well as HGSOC patients. *FOXP1* was detected as a partner gene in two recurrent combinations with *MALAT1* (2/20, 10%) and was additionally observed in two additional fusions. The frequency of all partner genes in recurrent and unique gene fusions is given in Supplementary Table 3

Among the 156 fusion genes, 44 (28%) events were confirmed to be recurrent in at least two patients (Table 3). This accounted for a mean of 2.4 ± 0.5 (± SEM, range 0–8) recurrent fusion events per patient. In total, 20 different recurrent fusion genes were observed. In the TNBC subgroup, a frequency of 36% (27/74 events) recurrent fusion events was detected with a mean of 3.0 ± 0.9 (± SEM, range 0–8) per patient. This compared to a lower rate of 21% recurrent fusion genes (17/82 events) in the HGSOC subgroup with a mean of 1.9 ± 0.5 (± SEM 0–4) per patient (Table 3). Intergroup comparison was not statistically significant (*P *= 0.5, Fig. 1b). Eleven of 20 (55%) recurrent fusion events were shared between TNBC and HGSOC patients (Fig. 2). Six fusion events were only detected in TNBC patients (*SPTAN1*–*MALAT1*, *RNF213*–*MALAT1*, *MALAT1*–*WWOX*, *MALAT1*–*FOXP1*, *MALAT1*–*DST*, *ATXN3*–*THAP11*), while three fusion genes were uniquely observed in HGSOC patients, only (*OGT*–*MUC16*, *NCL*–*MUC16*, *MUC16*–*NCL*, Fig. 2). Four fusion events (*WWOX*–*MALAT1, THAP11*–*ATXN3*, *SMG1*–*MALAT1*, *MALAT1*–*VPS13B*) occured in a total of three different patients each.

**Table 3:** Recurrent fusions

Number of fusions	*n*	% of total
All patients (*n *= 18)	156	100
Recurrent	44	28
Non-recurrent	111	71
HGSOC (*n *= 9)	82	53
Recurrent of total HGSOC	17	21
Non-recurrent of total HGSOC	65	79
TNBC (*n *= 9)	74	47
Recurrent of total TNBC	27	36
Non-recurrent of total TNBC	47	64

### MALAT1, MUC16, FOXP1, WWOX and XIST are the most common genes partnering in gene fusions

We next examined our dataset to look for genes repeatedly involved in gene fusions. Among the five most frequently detected genes, the long non-coding RNA (lncRNA) *MALAT1* was involved in 62% (97/156 events, 13 different individuals) of fusion transcripts, followed by the protein-coding mucin family gene *MUC16* (12%, 19/156 events, three different individuals) and *FOXP1* coding for the tumor suppressor and forkhead box transcription factor protein 1 (4%, 6/156 events, five different individuals). The tumor suppressor gene *WWOX* and the non-protein-coding X inactive-specific transcript (*XIST*) were detected in 6/156 (4%) events and four different individuals each. Table 4 illustrates the frequency of *MALAT1*, *MUC16*, *FOXP1, WWOX* and *XIST* across TNBC and HGSOC patients. A detailed overview of all detected fusion gene partners and their frequency is given in Supplementary Table 3.

**Table 4:** The top three partner genes involved in fusions

Number of fusions	*n*	%	Patients with respective fusion (%)
All fusions	156	100%	
*MALAT1*	97	62%	
TNBC	55	57% of *MALAT1*	6/9 (66%)
HGSOC	42	43% of *MALAT1*	7/9 (78%)
*MUC16*	19	12%	
TNBC	0	0% of *MUC16*	0/9 (0%)
HGSOC	19	100% of *MUC16*	3/9 (33%)
*FOXP1*	6	4%	
TNBC	5	83% of *FOXP1*	4/9 (44%)
HGSOC	1	16% of *FOXP1*	1/9 (11%)
*WWOX*	6	4%	
TNBC	4	66% of *WWOX*	2/9 (22%)
HGSOC	2	33% of *WWOX*	2/9 (22%)
*XIST*			
TNBC	3	50% of *XIST*	2/9 (22%)
HGSOC	3	50% of *XIST*	2/9 (22%)

*MALAT1* was detected as partner gene in both subgroups but was more prevalent in TNBC (57%) as compared to HGSOC patients (43%). Fusion genes involving *MUC16* were exclusively observed in HGSOC patients accounting for 22% (19/82 events) of all fusion transcripts detected in the HGSOC subgroup. Similar to *MALAT1*, *FOXP1* was detected as a partner gene in both subgroups, noting a higher prevalence in the TNBC subgroup (83% of all *FOXP1* fusion events). *WWOX* and *XIST* fusions were both identified in two TNBC and two HGSOC patients (Fig. 4).

**Fig. 3:**
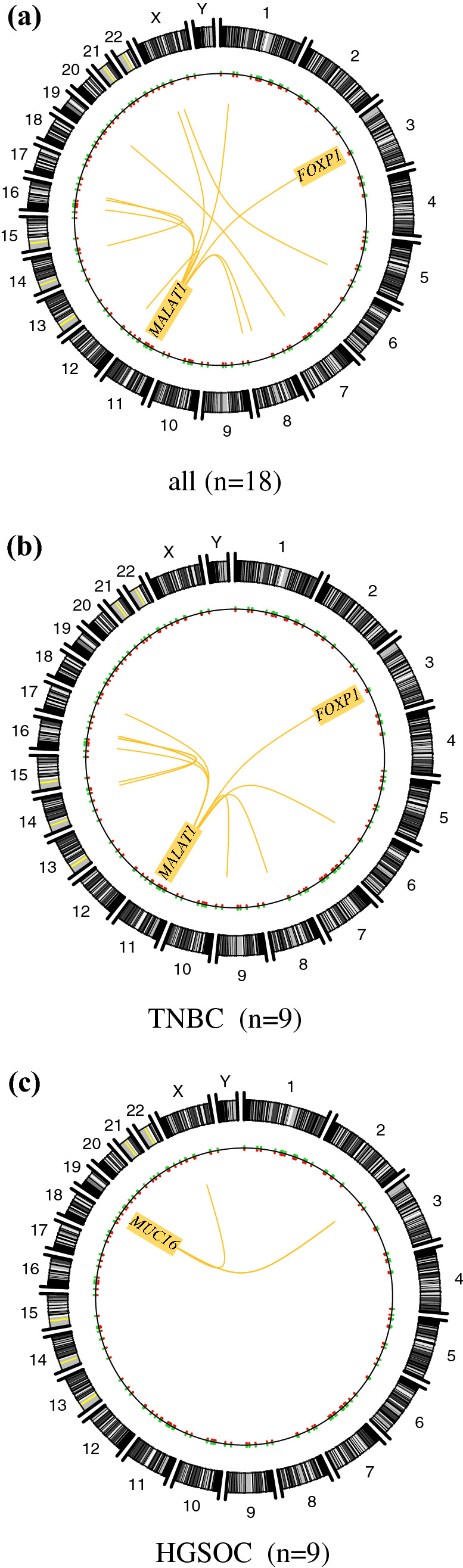
Distribution of fusion genes across the genome. Circos plots illustrating all recurrent fusion rearrangements in all patients (**a***n *= 18), in TNBC patients (**b***n *= 9) and HGSOC patients, only (**c***n *= 9). The predominance of fusion genes involving chromosome 11 was attributable to fusion transcripts involving the lncRNAs *MALAT1* located on this chromosome. *MALAT1* (chromosome 11) was highly promiscuous and formed recurrent fusions with numerous partner genes from a number of chromosomes, thereby resulting in a highly complex chromosomal distribution in TNBC patients. The chromosomal distribution for HGSOC was less abundant than that for TNBC and was mostly limited to chromosome 19 (*MUC16*) and nearby partner chromosomes (chr X, 2). Similar to *MALAT1*, *FOXP1* was detected as a partner gene in recurrent fusion genes in both subgroups, noting a higher prevalence in the TNBC subgroup (83% of all *FOXP1* fusion events)

Circos plots were used to illustrate and compare gene fusion combinations that were recurrently shared in TNBC and HGSOC patients (Fig. 3a) as compared to TBNC (Fig. 3b) and HGSOC (Fig. 3c) patients, only. *MALAT1* (chr 11) was highly promiscuous and formed recurrent fusions with numerous partner genes from a number of chromosomes, including chromosomes 6, 8, 9, 14, 16, 17, and *FOXP1* on chromosome 3 (Fig. 3b). In HGSOC, the fusion landscape was dominated by *MUC16* on chromosome 19 which partnered with genes on nearby partner chromosomes (chr X and 2: Fig. 3c).

### Differential gene expression in three fusion gene partners

Upregulation of *ABL1* gene expression is a common feature of *BCR*–*ABL*1-positive versus -negative acute lymphoblastic leukemia (Juric et al. 2007). Following this trajectory, we aimed to determine whether gene fusions in our dataset would also result in differential expression of the respective genes involved. A total of 20 recurrent fusion genes were detected. One of these partner genes (*C19MC*) was not annotated and thus excluded from the analysis.

**Fig. 4:**
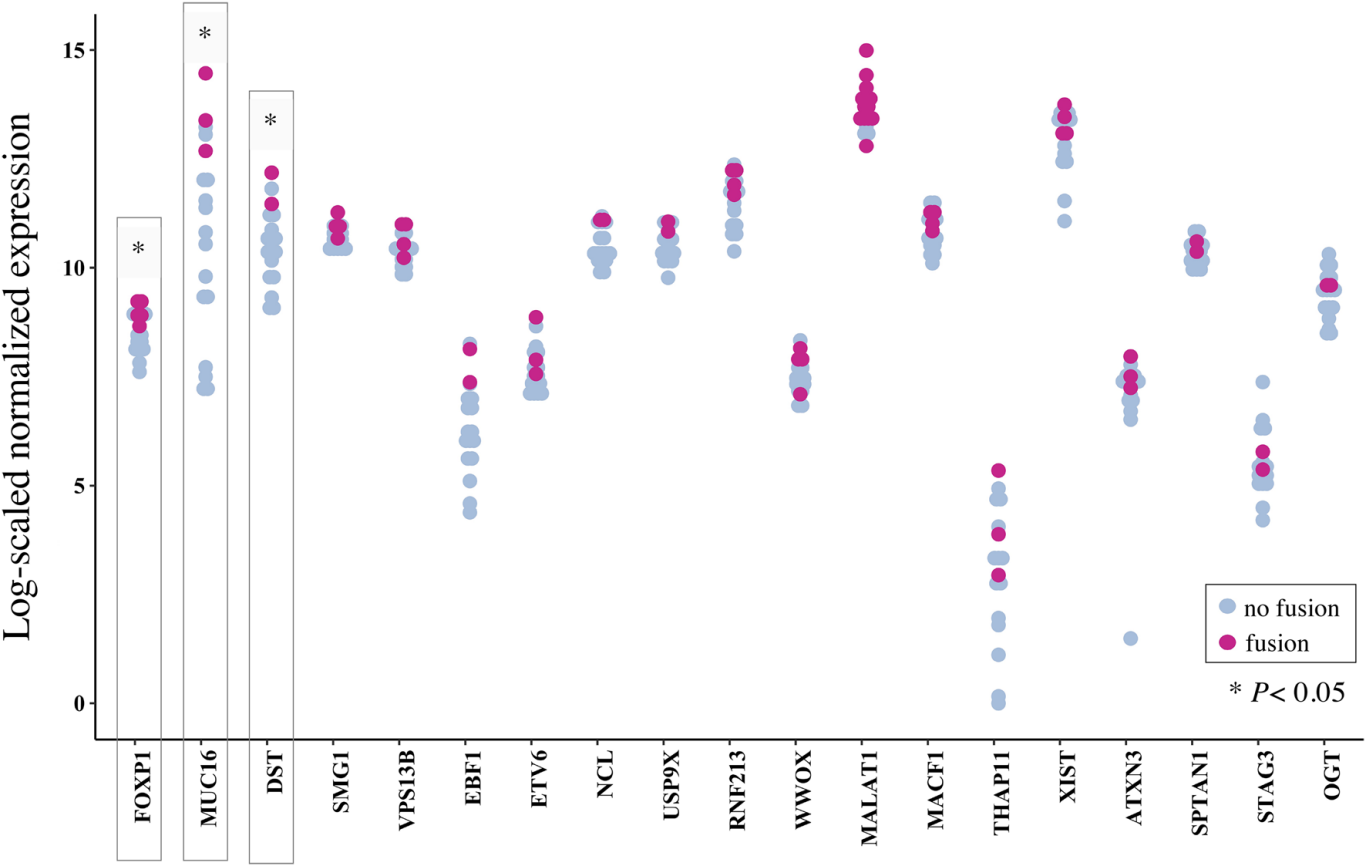
Differential gene expression in fusion partner genes. Scatter plot examining differential gene expression results from transcriptome analysis by DESeq2 in fusion-positive as compared to fusion-negative patients. A total of 20 genes were detected as recurrent fusion gene partners across our cohort. One fusion partner (*C19MC*) was not annotated and thus excluded from the analysis. Normalization of counts was performed separately for every gene for the combined cohort of TNBC and HGSOC patients (*n *= 18). *MALAT1* showed no significantly altered expression in patients who carried a fusion transcript with the respective gene involved vs. those without (*P* value = 0.29), whereas significant overexpression of *FOXP1* (*P *= 0.02), *MUC16* (*P *= 0.02) and *DST* (*P *= 0.02) was noted in patients with the respective fusion transcript as compared to controls

For the remaining 19 recurrent fusion partner genes, we performed differential gene expression analysis for fusion-positive versus -negative patients using *DESeq2*. Gene expression was significantly different in the three genes *FOXP1* (*P *= 0.02), *MUC16* (*P *= 0.02) and *DST* (*P *= 0.02) (Fig. 4, Table 5).

**Table 5:** Differential gene expression in samples with fusion versus no fusion

Gene	*P* value	FDR	log2FC
*FOXP1*	0.02*	0.20	0.75
*MUC16*	0.02*	0.20	2.28
*DST*	0.02*	0.22	1.60
*SMG1*	0.05	0.35	0.41
*VPS13B*	0.06	0.38	0.53
*EBF1*	0.09	0.50	1.38
*ETV6*	0.11	0.54	0.75
*RTATNCL*	0.11	0.55	0.74
*USP9X*	0.18	0.77	0.53
*RNF213*	0.19	0.78	0.55
*WWOX*	0.21	0.83	0.43
*MALAT1*	0.29	1.00	0.44
*MACF1*	0.31	1.00	0.34
*THAP11*	0.41	1.00	− 0.36
*XIST*	0.42	1.00	0.39
*ATXN3*	0.44	1.00	0.47
*SPTAN1*	0.63	1.00	0.16
*STAG3*	0.68	1.00	− 0.31
*OGT*	0.81	1.00	0.14

*FDR* false discovery rate, *log2FC* log2 fold change

Interestingly, although *MALAT1* was the most common partner gene, its expression was not significantly altered in tumors with and without fusions (*P *= 0.29). Similarly, expression of *WWOX* (*P *= 0.21) and *XIST* (*P *= 0.42) was not significantly different in tumors with versus without involvement of the respective fusion gene.

### Patients with FOXP1 fusion genes show significant FOXP1 overexpression and are associated with favorable overall survival

By performing differential gene expression analysis, we were able to demonstrate a functional impact of *MUC16*, *DST* and *FOXP1* fusions on the expression of the respective gene. In a final step, we examined the clinical variables associated with these transcripts.

*MUC16* codes for CA-125, an established serum marker for ovarian cancer in the clinic (Panza et al. 1988). In our cohort, *MUC16* fusion transcripts were exclusively detected in HGSOC patients. Since *MUC16* fusion genes could thus not provide a shared value for our entire study cohort, this gene was excluded from further correlation analyses. Similarly, fusion genes involving *DST* were deprioritized given the low frequency of *DST* fusion genes of only 2/18 (11%) patients which did not allow for relevant association with clinical endpoints.

**Fig. 5:**
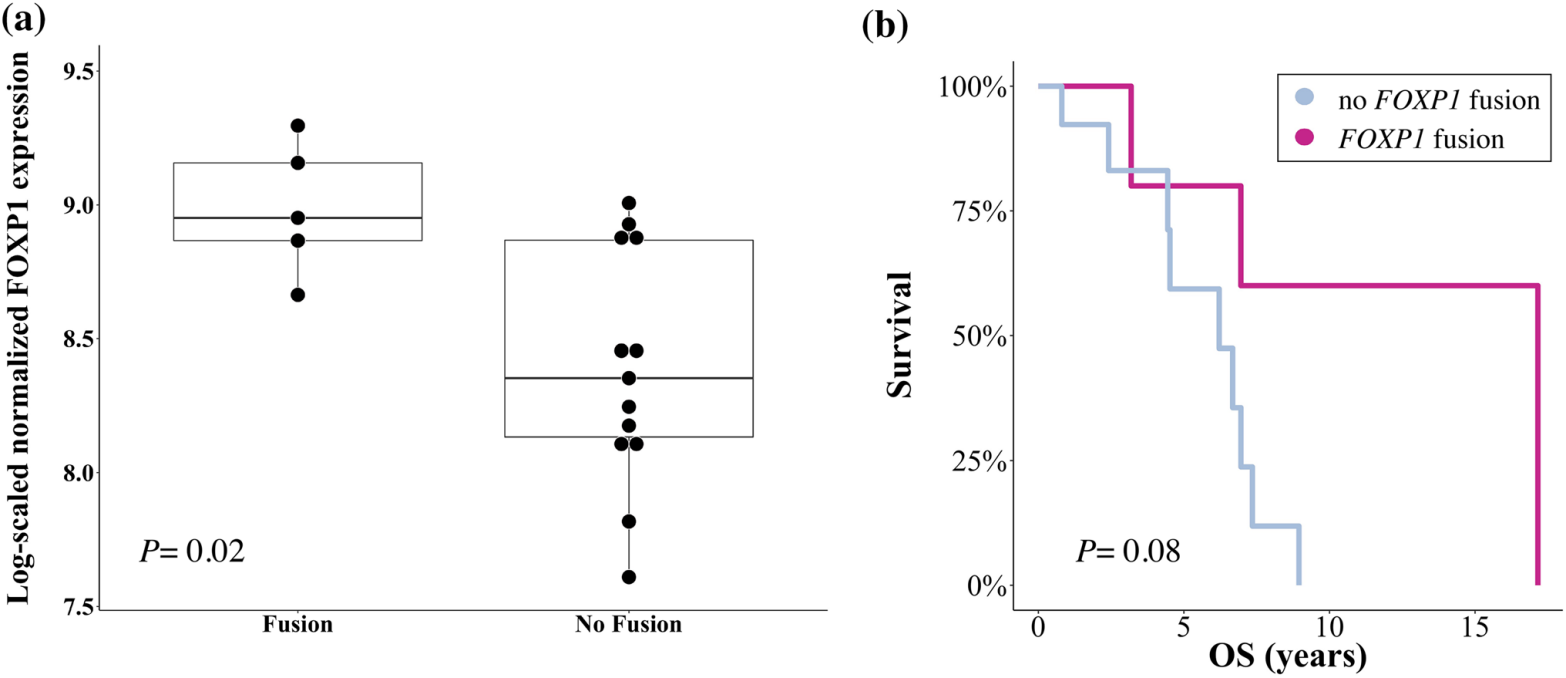
Correlation of *FOXP1* fusion genes with gene expression and overall survival. Box plots illustrating the expression level of *FOXP1* in TNBC and HGSOC patients with vs. without identified *FOXP1* fusion genes. Significant overexpression was noted when *FOXP1* was involved in a fusion gene (**a***P *= 0.02). Kaplan–Meier estimators were calculated to evaluate the overall survival (OS, years) in patients with as compared to patients without *FOXP1* fusion (**b**). For the calculation of OS, all patients alive at the time of this study were included as censored subjects. Patients lost to follow-up were censored at the last day confirmed alive. Superior survival was observed in fusion-positive patients. Given the small sample size of the here-presented study, this finding did not reach statistical significance (Mann–Whitney–Wilcoxon *P *= 0.08)

Consequently, we focussed on the clinical comparison analysis of *FOXP1* fusion genes for which differential gene expression was the most significant in patients with versus without a respective fusion (Fig. 5a, *P *= 0.02).

To estimate the prognostic impact of *FOXP1* fusion transcripts on the outcome of TNBC and HGSOC patients, we performed Kaplan–Meier estimators for *FOXP1* fusion-negative versus -positive patients. Median PFS on buparlisib/olaparib treatment was 12.1 months (95% CI 0.3–2.6) for patients with *FOXP1* fusions as compared to 13.9 months (95% CI 0.4–3.4) in those without (*P *= 0.97, Supplementary Fig. 2). This lack of significance was expected since no direct association between the *FOXP1* tumor suppressor gene and PI3K/PARP inhibition has been reported thus far. In a next step, we performed Kaplan–Meier estimators to determine whether *FOXP1* fusions were associated with a difference in OS. Median OS in *FOXP1* fusion-positive patients was 17.2 years (95% CI 0.8–10.2) as compared to a median OS of 6.2 years (95% CI 0.1–1.3) in *FOXP1* fusion-negative patients (Fig. 5b). As inherent to the small sample size of this study, this OS difference was not significant (*P *= 0.08) and interpretations need to remain exploratory.

## Discussion

In this study, transcriptome sequencing was applied to identify gene fusions in a cohort of 18 TNBC and HGSOC patients. Fusion events were detected by the use of the FusionCatcher algorithm, a tool validated to confirm true-positive gene fusions with reported filtering rates of ~ 40% (Nicorici et al. 2014; Engqvist et al. 2018; Parris et al. 2018). To further minimize the number of false-positive events in our analysis, we applied additional filtering steps. In total, this resulted in a number of 156/792 (20%) fusions which were kept for further exploration. This number compares to a similar filtering strategy reported in a 2018 study by Fimereli and colleagues, which reported a fraction of 316/1222 (26%) true fusion events identified by the deFuse algorithm with Ensemble release 62 (reference genome hg19, 22).

The mean number of fusion transcripts per patient in our study was reported at 8.7 ± 1.9 (± SEM). The prevalence of fusion genes was not significantly different between both tumor types (*P *= 0.62). Previous reports using FusionCatcher have reported a mean rate of 132.7 ± 31.0 (± SEM, range 12–613) and 34.7 ± 4.4 (± SEM, range 0–266) fusion events per sample in breast and ovarian cancers, respectively (Engqvist et al. 2018; Parris et al. 2018). It must be noted that the cohorts in those studies were not exclusively restricted to TNBC and HGSOC and are thus not entirely comparable to our analysis. Similarly, Fimereli and colleagues did not preselect their breast cancer cohort for TNBC patients and observed that the number of fusion genes per sample ranged between 0 and 31 with a mean of 6.7 fusion genes, noting the highest prevalence in HER2-positive tumors, which were not part of our study (Fimereli et al. 2018).

Overall, 109/156 (68%) fusion genes in our study had one gene partner with a non-CDS, but only 7/156 (4%) were confirmed to be CDS-complete. Engqvist and colleagues recently reported a comparable rate of 76% non-CDS fusion genes in a cohort of 96 early-stage ovarian cancer patients (Engqvist et al. 2018). A similar rate of 86% non-CDS fusion genes has been reported by Parris and colleagues for a cohort of 185 breast cancer patients (Parris et al. 2018). Our data thus suggest that non-CDS fusion gene partners seem to be similarly prevalent in TNBC and HGSOC as previously confirmed for the aforementioned unselected populations of breast and ovarian cancers.

It has been described that the rate of recurrent fusion genes in breast and ovarian cancers is low and may be even more limited in tumors with high genomic instability (Mertens et al. 2015; Yoshihara et al. 2015). Correspondingly, only 44/156 (28%) fusion genes in our cohort were detected in more than two patients. None of these fusion genes have been previously detected in the TCGA cohort for breast and ovarian cancers (Yoshihara et al. 2015). At the same time, 59/88 (67%) of the individual genes we identified had also been reported by Yoshihara and colleagues in their extensive interrogation of 4366 tumor samples (Yoshihara et al. 2015). Our analysis thus supports prior studies indicating that the majority of gene fusions in epithelial cancers are most likely private passenger events with low recurrence across samples (Mertens et al. 2015).

The majority of fusion genes in our study involved at least one partner gene located on chromosome 11. Such chromosomal hotspots for fusion genes have been reported. The study by Fimereli and colleagues has observed fusion hotspots in breast cancer on chromosomes 17, 8 and 20 (Fimereli et al. 2018). Similar to our observation, the majority of fusion transcripts in the studies by Engqvist et al. (2018) and Parris et al. (2018) equally involved chromosome 11 as predominant fusion hotspot. This striking relationship was mostly attributable to fusion transcripts involving the lncRNA *MALAT1* located on chromosome 11.

Long non-coding RNAs are defined by having a length exceeding 200 nucleotides (Mendell 2016). The human genome encodes many thousands of these lncRNAs but their role in cancer remains to be comprehensively characterized. *MALAT1* was one of the first human lncRNAs to be discovered in samples of metastatic lung cancer cells (Ji et al. 2003). Since then, it has been shown to be associated with metastasis and poor survival in multiple malignancies including breast cancer (Gutschner et al. 2013). Its exact molecular function, however, still remains poorly understood. *MALAT1* fusion genes were previously detected in breast cancer and ovarian cancer samples (Engqvist et al. 2018; Parris et al. 2018). In these samples, *MALAT1* was determined to be highly promiscuous with over 400 partner genes, indicating that the majority of *MALAT1* fusions may occur at the RNA level (Parris et al. 2018).

In our cohort, *MALAT1* was involved in 97/156 (62%) fusion genes and partnered with 68 different gene partners. 55/74 (74%) fusion genes in TNBC and 42/82 (51%) fusion genes in HGSOC involved *MALAT1* as one of their partnering genes. Overall, *MALAT1* fusions were detectable in six TNBC and seven HGSOC patients. Since lncRNAs do not generate a corresponding fusion protein but may influence the expression of the respective fusion partner, further research is necessary to elucidate their role for tumor formation.

The expression of partner genes involved in gene fusions may be substantially altered once fused to a partner gene with promoter activity (Juric et al. 2007). To examine how fusion genes affected differential gene expression of both partner genes in our cohort, we next performed differential gene expression analysis for the 19 most common fusion partner genes in our cohort. Among these, *FOXP1*, *MUC16* and *DST* showed a significantly altered expression in those patients who carried a respective fusion transcript as compared to those without. Notably, and in contrast to prior reports in ovarian cancer, no such relationship was observed for *MALAT1* (Engqvist et al. 2018).

*MUC16* is a mucin family gene that codes for Cancer Antigen 125 (CA-125). CA-125 has been used to monitor ovarian cancer in the clinic for many years (NIH consensus conference 1995). Expectedly, *MUC16* was determined to be a frequent driver fusion transcript in the ovarian cancer study by Engqvist and colleagues (Engqvist et al. 2018) and was similarly prevalent in 3/9 (33%) of HGSOC patients in our cohort.

The most notable candidate gene in our study, however, *FOXP1,* was involved in fusion genes of a total of four TNBC patients (one case with two detectable *FOXP1* fusion genes) and one HGSOC patient. The presence of *FOXP1* fusion genes corresponded to a significant overexpression of *FOXP1*. Correlation studies with clinical endpoints need to remain exploratory, mostly since only one HGSOC patient was tested positive for carrying a *FOXP1* fusion gene. However, the median OS in *FOXP1* fusion positive patients was 17.2 years as compared to a median OS of 6.2 years in *FOXP1* fusion negative patients (*P *= 0.08). The potential role of *FOXP1* as a prognostic marker in oncology remains controversial. High *FOXP1* expression has previously been linked to metastasis and poor five-year OS in a cohort of 101 non-small cell lung cancer patients (Feng et al. 2012). At the same time, other studies have reported an association between *FOXP1* overexpression and inferior outcome in the context of hematologic malignancies such as follicular lymphoma and diffuse-large B-cell lymphoma (Mottok et al. 2018; Barrans et al. 2004).

Our observation may be in line with previous studies in breast cancer that observed high *FOXP1* protein expression to be a favorable prognostic marker in patients with estrogen receptor (ER)-positive breast cancer (Bates et al. 2008; Fox et al. 2004; Rayoo et al. 2009). Our analysis expands the scope of these latter studies by investigating the presence of *FOXP1* fusion genes in TNBC and HGSOC. Notably, as 83% of the identified *FOXP1* fusion genes in our cohort have been detected in TNBC patients, we conclude that *FOXP1* may act as a tumor suppressor independently of ER expression. The correlation detected in this study should be treated with caution given the small sample size inherent to a phase I study. Similarly, the comparison between fusion-positive (*n *= 5) and fusion-negative (*n *= 13) patients was not balanced in numbers and thus biased for a greater variation (*P *= 0.02). More comprehensive analyses will be needed to confirm the prognostic value of *FOXP1* fusion genes.

The limited sample size in our study remains an inevitable shortcoming of this analysis. Tumor tissue from initial diagnosis was only available for 18/69 patients of the entire study cohort meaning that our analysis might not have been powered to detect significant differences. It must also be noted that, despite its merits and meticulous filtering, RNA-seq may not detect some fusion genes including those involving non-transcribed enhancer or promoter elements (Kim and Salzberg 2011). Future approaches will thus have to complement transcriptome analysis by whole-genome-sequencing and RT-PCR.

In summary, our analysis provides the first comprehensive analysis of the fusion gene landscape in a homogeneously treated cohort of TNBC and HGSOC patients. We provide evidence for the low frequency of recurrent fusion genes in both cancer types. The lncRNA *MALAT1* was a highly prevalent fusion partner in our analysis, but larger studies will have to further determine its potential as prognostic biomarker in TNBC and HGSOC. Interestingly, three fusion gene partners showed a significantly altered expression in patients carrying the respective fusion. Among these, *FOXP1* fusions seem to be associated with a favorable prognosis in TNBC and HGSOC patients. Such observations may help to increase our understanding on the role of fusion genes in female cancer. This seems particularly relevant for cancers with limited treatment options such as TNBC and HGSOC, for which a better mechanistic understanding of how fusion genes interfere with functional gene expression may provide vital clues to finding new and innovative therapeutic strategies.

## Electronic supplementary material

Below is the link to the electronic supplementary material.

Supplementary material 1 (PDF 382 kb)
