# Supplementary material for: Transcriptome analysis reveals overlap in fusion genes in a phase I clinical cohort of TNBC and HGSOC patients treated with buparlisib and olaparib
Source: J Cancer Res Clin Oncol. 2019 Nov 19;146(2):503–14. doi: 10.1007/s00432-019-03078-9 (PMC6985087; doi:10.1007/s00432-019-03078-9)

Original Article

# Transcriptome analysis reveals overlap in fusion genes in a phase I clinical cohort of TNBC and HGSOC patients treated with buparlisib and olaparib

**Running Title:** Transcriptome and fusion gene landscape in TNBC and HGSOC

Julia Eismann[1,2,3], Yujing J. Heng[2,4], Johannes M. Waldschmidt[2,5], Ioannis S. Vlachos[2,4], Kathryn Gray[2,6], Ursula Matulonis[2,5], Panos A. Konstantinopoulos[2,5], Charles J. Murphy[7], Sheida Nabavi[8], Gerburg Wulf[1,2]

**Affiliations:**

[1] Department of Hematology/Oncology, Beth Israel Deaconess Medical Center, Boston, MA
[2] Harvard Medical School, Boston, MA
[3] Department of Obstetrics and Gynecology, University Medical Center Freiburg, Freiburg, Germany
[4] Department of Pathology, Beth Israel Deaconess Medical Center, Boston, MA
[5] Department of Medical Oncology, Dana-Faber Cancer Institute, Boston, MA
[6] Biostatic Core, Dana Faber, Harvard Cancer Center, Boston, Massachusetts
[7] Institute for Computational Biomedicine, Weill Cornell Medical College, New York, New York
[8] Department of Computer Science and Engineering, Institute of System Genomics, University of Connecticut

**Correspondence:** Gerburg Wulf, MD, PhD, e-mail: gwulf@bidmc.harvard.edu

**Key words:** fusion gene, breast cancer, ovarian cancer, genomic profiling, RNA-seq

Formatted for
**Journal of Cancer Research and Clinical Oncology**

*Supplementary Figures and Tables*
*(please see attached image files for high-resolution figures)*

**Supplementary Table 1. Distribution of fusions before correction for false-positives**

| Detected Fusions (applied filters) | | number | % of total |
|---|---|---|---|
| **Total number of fusions detected** | | **792** | **100%** |
| Fusion Description | Readthrough | 17 | 2% |
| | Non-tumor-cell | 324 | 41% |
| | Ribosomal | 1 | 0.1% |
| | Adjacent | 13 | 1.6% |
| | Pseudogene | 4 | 0.5% |
| | Immunoglobulin | 10 | 1.2% |
| | Breakpoint within homologous polyglutamine repeat regions | 5 | 0.6% |
| **Total number of fusions corrected for false-positives** | | **418** | **53%** |
| | Unique | 156 | 20% |
| | Protein coding | 47 | 6% |
| | No protein | 109 | 14% |

**Supplementary Table 2. Distribution of the predicted effect of fusion transcripts across the study cohort**

| Predicted function | n | % of total |
|---|---|---|
| Total number of corrected fusion genes | 156 | 100% |
| no CDS/ protein | 109 | 68% |
| truncated | 19 | 12% |
| in-frame | 10 | 6% |
| out-of-frame | 3 | 2% |
| other (intronic/CDS complete) | 7 | 4% |
| unknown | 8 | 5% |

**Supplementary Table 3. Overall frequency of detected fusion gene partners in study cohort (n=312 partner genes)**

| Gene partner | TNBC (n) | HGSOC (n) | Patients (n) |
|---|---|---|---|
| *ACSL3* | 1 | | 1 |
| *ACTB* | 3 | | 1 |
| *AHNAK* | 1 | 2 | 3 |
| *AKAP13* | 1 | | 1 |
| *ALK* | | 2 | 1 |
| *ANKMY1* | | 1 | 1 |
| *ANKRD11* | | 1 | 1 |
| *ANTXR1* | | 1 | 1 |
| *AP005135.2* | 1 | | 1 |
| *APBB2* | 1 | | 1 |
| *APP* | 2 | | 2 |
| *ARMC9* | | 1 | 1 |
| *ASH1L* | 1 | | 1 |
| *ATM* | 1 | | 1 |
| *ATP11B* | 2 | | 2 |
| *ATXN3* | 4 | 1 | 3 |
| *BBX* | | 1 | 1 |
| *BCL2* | 1 | | 1 |
| *BIRC6* | 1 | | 1 |
| *BMP2K* | | 1 | 1 |
| *C19MC* | 1 | 1 | 2 |
| *C1ORF186* | | 1 | 1 |
| *C2ORF81* | | 1 | 1 |
| *CBWD2* | | 1 | 1 |
| *CCDC102B* | 1 | | 1 |
| *CCDC6* | | 1 | 1 |
| *CD47* | | 1 | 1 |
| *CD9* | | 1 | 1 |
| *CDH1* | | 1 | 1 |
| *CHD6* | | 1 | 1 |
| *CHST11* | | 1 | 1 |
| *CNOT1* | | 1 | 1 |
| *COL11A1* | | 2 | 1 |
| *COL14A1* | | 1 | 1 |
| *COL1A1* | | 1 | 1 |
| *COL1A2* | | 1 | 1 |
| *COL3A1* | 1 | | 1 |
| *COL6A2* | 1 | | 1 |
| *DMD* | | 1 | 1 |
| *DNM1L* | 1 | | 1 |
| *DPYD* | 1 | | 1 |
| *DST* | 2 | | 2 |
| *EBF1* | 1 | 1 | 2 |
| *EEF1A1* | | 2 | 2 |
| *EP300* | 1 | | 1 |
| *ERP44* | | 1 | 1 |
| *ETV6* | 2 | 2 | 3 |
| *FN1* | | 1 | 1 |
| *FNDC3B* | 1 | | 1 |
| *FOXO6* | | 1 | 1 |
| ***FOXP1*** | **5** | **1** | **5** |
| *GAPDH* | | 1 | 1 |
| *GSN* | | 1 | 1 |
| *HIST1H2AI* | 1 | | 1 |
| *HIST3H2A* | 1 | | 1 |
| *HMGA1* | | 1 | 1 |
| *HMOX1* | 1 | | 1 |
| *HNRNPK* | 1 | | 1 |
| *HUWE1* | | 1 | 1 |
| *IGF1R* | 1 | | 1 |
| *IGF2* | | 1 | 1 |
| *ILF3* | 1 | | 1 |
| *IQCK* | | 1 | 1 |
| *IQGAP1* | 1 | | 1 |
| *IRF2BPL* | | 1 | 1 |
| *ITPR2* | 1 | | 1 |
| *KIAA1217* | | 1 | 1 |
| *KIDINS220* | | 1 | |

| Gene partner | TNBC (n) | HGSOC (n) | Patients (n) |
|---|---|---|---|
| *KMT2C* | | 1 | 1 |
| *LAMA4* | | 1 | 1 |
| *LAMB1* | 1 | | 1 |
| *LINC00578* | | 1 | 1 |
| *LMO7* | | 1 | 1 |
| *LPP* | | 1 | 1 |
| *MACF1* | 3 | 1 | 4 |
| ***MALAT1*** | **55** | **42** | **13** |
| *MAML2* | | 1 | 1 |
| *MAP4* | | 1 | 1 |
| *MED12* | | 1 | 1 |
| *MED13L* | 1 | | 1 |
| *MLPH* | 1 | | 1 |
| *MSI2* | 1 | | 1 |
| *MTOR* | | 1 | 1 |
| *MTR* | | 1 | 1 |
| ***MUC16*** | | **19** | **3** |
| *MYCBP2* | | 1 | 1 |
| *MYH11* | 1 | | 1 |
| *MYLK* | | 1 | 1 |
| *NCKAP1L* | 1 | | 1 |
| *NCL* | | 4 | 2 |
| *NEAT1* | | 1 | 1 |
| *NFIA* | | 1 | 1 |
| *OGT* | | 2 | 2 |
| *PAM* | | 1 | 1 |
| *PAN3* | 1 | | 1 |
| *PDIA3* | | 1 | 1 |
| *PLCB1* | | 1 | 1 |
| *PLEC* | | 1 | 1 |
| *PLXDC2* | | 1 | 1 |
| *POLA1* | | 1 | 1 |
| *PRRC2C* | 1 | | 1 |
| *PSD4* | | 1 | 1 |
| *PSPC1* | 1 | | 1 |
| *PUS3* | | 1 | 1 |
| *RBM25* | | 1 | 1 |
| *RBMS1* | 2 | | 1 |
| *RMRP* | 2 | 1 | 2 |
| *RNF213* | 3 | 1 | 4 |
| *SETX* | | 1 | 1 |
| *SF3B3* | 1 | | 1 |
| *SMG1* | 4 | 1 | 4 |
| *SPTAN1* | 2 | | 2 |
| *SPTBN1* | | 1 | 1 |
| *SREBF2* | | 1 | 1 |
| *STAG3* | 1 | 1 | 2 |
| *SULF1* | | 1 | 1 |
| *SYNE1* | | 1 | 1 |
| *SYNE2* | | 1 | 1 |
| *TBC1D1* | | 1 | 1 |
| *THAP11* | 4 | 1 | 3 |
| *THRAP3* | | 1 | 1 |
| *TIMP3* | | 1 | 1 |
| *USP9X* | 1 | 1 | 2 |
| *VMP1* | 1 | | 1 |
| *VPS13B* | 3 | 2 | 4 |
| *WDR26* | 1 | | 1 |
| *WDR43* | | 1 | 1 |
| ***WWOX*** | **4** | **2** | **4** |
| ***XIST*** | **3** | **3** | **4** |
| *XXBAC* | | 1 | 1 |
| *YBX1* | 1 | | 1 |
| *YLPM1* | 1 | | 1 |
| *ZNF124* | | 1 | 1 |
| *ZNF664* | | 1 | 1 |
| *ZNF843* | | 1 | 1 |
| *ZNFX1* | 1 | | 1 |

## Supplementary Table 4. Patient characteristics in *FOXP1* fusion-positive versus -negative patients (n=18)

| Variables | *FOXP1* fusion positive (n=5) | *FOXP1* fusion negative (n=13) |
|---|---|---|
| **Age at diagnosis** | | |
| years (mean ± SEM [range]) | 55 ± 5.1 (44-70) | 56 ± 2.9 (36-72) |
| **Age at inclusion** | | |
| years (mean ± SEM [range]) | 60 ± 4.0 (49-70) | 59 ± 2.9 (38-78) |
| **Race** | | |
| White | 5/5 (100%) | 13/13 (100%) |
| **Ethnicity** | | |
| Hispanic or Latino | 1 (20%) | 1 (8%) |
| non-Hispanic | 3 (60%) | 12 (92%) |
| unknown | 1 (20%) | |
| **Platinum status** | | |
| platinum resistant | 2 (40%) | 5 (38%) |
| platinum sensitive | 1 (20%) | 4 (31%) |
| unknown | 2 (40%) | 4 (31%) |
| **Stage** | | |
| I | 2 (40%) | 3 (23%) |
| II | 1 (20%) | 7 (54%) |
| III | 2 (40%) | 3 (23%) |
| IV | | |
| **Histology** | | |
| adenocarcinoma | 2 (40%) | 2 (15%) |
| papillary serous | | 8 (62%) |
| transitional | 1 (20%) | |
| others | 2 (40%) | 3 (23%) |
| **Clinical Grade** | | |
| moderately differentiated | 1 (20%) | 2 (15%) |
| poorly differentiated | 4 (80%) | 11 (85%) |
| **Prevalence of fusion genes** | | |
| mean ± SEM [range] | 4.8 ± 2.6 (0-11) | 9.9 ± 2.4 (0-21) |
| **PFS** | | |
| months (median, 95% CI) | 12.1 (0.3-2.6) | 13.4 (0.4-3.4) |
| **Reason for discontinuation** | | |
| progression by RECIST 1.1 | 5 (100%) | 11 (85%) |
| unacceptable toxicity | | 2 (15%) |
| **Overall survival** | | |
| years (median, 95% CI) | 17.2 (0.8-10.2) | 6.2 (0.1-1.3) |

**Supplementary Figure 1. Frequency of fusion genes relative to clinical characteristics and subtypes.** Box plots were used to illustrate the number of fusions at initial diagnosis per age group (a), per UICC stage (b), per differentiation grade (c) and relative to *BRCA* mutation status (d). No significant correlation was noted for either of the latter categories (Kruskal-Wallis test *P*=0.63, *P* =0.66, *P*=0.86 and *P* =0.28, respectively)

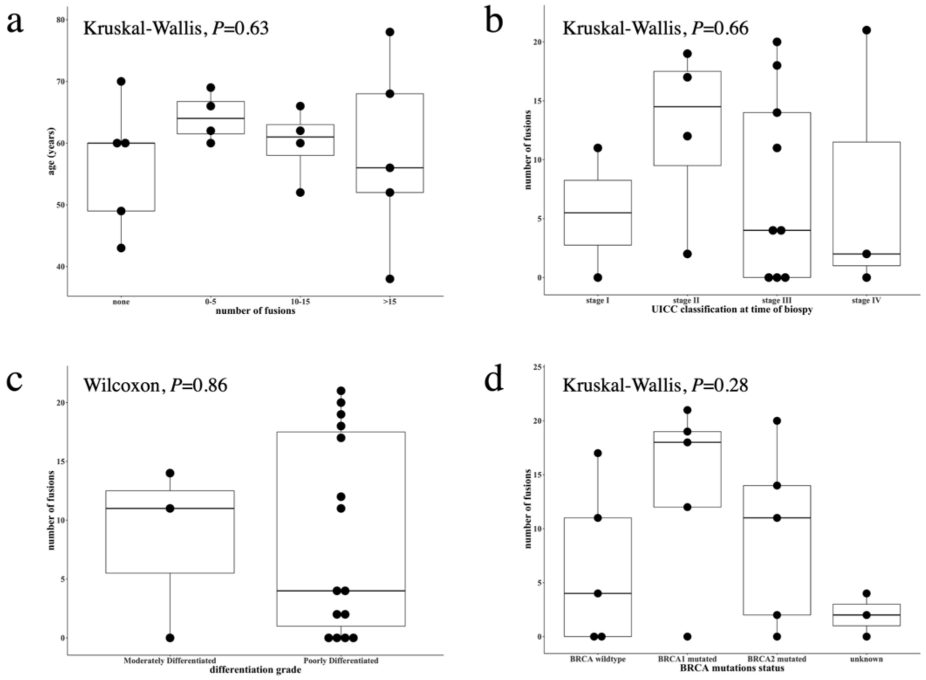

**Supplementary Figure 2. Correlation of *FOXP1* fusion genes with progression-free survival.** Kaplan-Meier estimators were calculated to evaluate the progression-free survival (PFS, months) on buparlisib/ olaparib treatment in TNBC and HGSOC patients with as compared to patients without detectable *FOXP1* fusion gene. All patients had relapsed at the time of data analysis. No trend was observed for superior PFS in *FOXP1* fusion-positive patients (Mann-Whitney-Wilcoxon *P*=0.97).

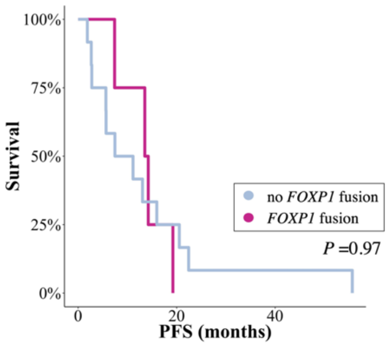

Supplement: Supplementary file 1 — Supplementary material 1 (PDF 382 kb) [file 432_2019_3078_MOESM1_ESM.pdf]
